# Supplementary material for: Ultrasound Can Be Usefully Integrated with the Clinical Assessment of Nail and Enthesis Involvement in Psoriasis and Psoriatic Arthritis
Source: J Clin Med. 2022 Oct 26;11(21):6296. doi: 10.3390/jcm11216296 (PMC9657153; doi:10.3390/jcm11216296)
Supplement: Supplementary file 1 [file jcm-11-06296-s001.zip › jcm-1919423-supplementary.pdf]

SUPPLEMENTARY DATA

Supplementary Table S1. Subtypes of nail psoriasis.

Parameters	PsA (n=154)	PsO(n=35)	p value
Nail matrix			
Pitting	61(39.6%)	20(57.1%)	0.067
Leukonychia	25(16.2%)	8(22.9%)	0.317
Red spots in the lunula	0(0%)	0(0%)	-
Crumbling	26(16.9%)	8(22.9%)	0.426
Nail bed			
Onycholysis	69(44.8%)	20(57.1%)	0.210
Splinter hemorrhage	18(11.7%)	8(22.9%)	0.089
Subungual hyperkeratosis	18(11.7%)	9(25.7%)	**0.035***
Oil stains	2(1.3%)	0(0%)	0.495

PsO, psoriasis; PsA, psoriatic arthritis; n, the number of patients

Supplementary Table S2. Independent predictors of fingernail thickness in patients with PsA and PsO.

Independent variables	t, adjusted OR (95%CI)	p
Age (years)	1.019 (0.989-1.051)	0.217
Gender (male vs. female)	1.136 (0.439-2.939)	0.739
Body mass index (kg/m2)	1.015 (0.916-1.125)	0.775
NAPSI	0.977 (0.959-0.995)	**0.013***
GUESS	1.332 (1.046-1.696)	**0.020***

PsO, psoriasis; PsA, psoriatic arthritis; NAPSI, nail psoriasis severity index; GUESS, Glasgow Ultrasound Enthesitis Scoring System.
